# Developing emergency care systems: a human rights-based approach

**DOI:** 10.2471/BLT.18.226605

**Published:** 2019-06-19

**Authors:** Taylor W Burkholder, Kimberly Hill, Emilie J Calvello Hynes

**Affiliations:** a Department of Emergency Medicine, University of Southern California, 1200 N State St Room 1011, Los Angeles, California 90033, United States of America (USA).; b Department of Emergency Medicine, Denver Health Medical Center, Denver, USA.; c Department of Emergency Medicine, University of Colorado School of Medicine, Denver, USA.

## Abstract

The delivery of emergency care is an effective strategy to reduce the global burden of disease. Emergency care cross cuts traditional disease-focused disciplines to manage a wide range of the acute illnesses and injuries that contribute substantially to death and disability, particularly in low- and middle-income countries. While the universal health coverage (UHC) movement is gaining support, and human rights and health systems are integral to UCH, few concrete discussions on the human right to emergency care have been taken place to date. Furthermore, no rights-based approach to developing emergency care systems has been proposed. In this article, we explore key components of the right to health (that is, availability, accessibility, acceptability and quality of health facilities, goods and services) as they relate to emergency care systems. We propose the use of a rights-based framework for the fulfilment of core obligations of the right to health and the progressive realization of emergency care in all countries.

## Résumé

La prestation de soins d'urgence constitue une stratégie efficace pour réduire la charge mondiale de morbidité. Les soins d'urgence recoupent les disciplines traditionnelles centrées sur les maladies pour prendre en charge de nombreuses blessures et affections aiguës qui contribuent sensiblement aux décès et aux handicaps, en particulier dans les pays à revenu faible et intermédiaire. Alors que le mouvement pour la couverture sanitaire universelle prend de l'ampleur et que les droits de l'homme et les systèmes de santé en font partie intégrante, peu de discussions concrètes sur le droit à des soins d'urgence ont eu lieu à ce jour. En outre, aucune démarche fondée sur les droits et visant à développer des systèmes de soins d'urgence n'a été proposée. Dans cet article, nous nous intéressons aux composantes clés du droit à la santé (à savoir la disponibilité, l'accessibilité, l'acceptabilité et la qualité des établissements, des produits et des services de soins) pour ce qui est des systèmes de soins d'urgence. Nous proposons d'utiliser un cadre fondé sur les droits pour l'exécution des obligations essentielles du droit à la santé et la mise en place progressive de soins d'urgence dans tous les pays.

## Resumen

La prestación de atención de emergencia es una estrategia eficaz para reducir la carga mundial de morbilidad. La atención de emergencia trasciende las disciplinas tradicionales centradas en las enfermedades para tratar una amplia gama de enfermedades y lesiones agudas que contribuyen sustancialmente a la muerte y la discapacidad, en particular en los países de ingresos bajos y medianos. Si bien el movimiento de la cobertura sanitaria universal (CSU) está ganando apoyo, y los derechos humanos y los sistemas de salud son parte integral de la CSU, hasta la fecha se han llevado a cabo pocas discusiones concretas sobre el derecho humano a la atención de emergencia. Además, no se ha propuesto un enfoque basado en los derechos para desarrollar sistemas de atención de emergencia. En este artículo exploramos los componentes clave del derecho a la salud (es decir, disponibilidad, accesibilidad, aceptabilidad y calidad de las instalaciones, bienes y servicios sanitarios) en relación con los sistemas de atención de emergencia. Proponemos el uso de un marco basado en los derechos para el cumplimiento de las obligaciones básicas del derecho a la salud y la realización progresiva de la atención de emergencia en todos los países.

## ملخص

إن تقديم الرعاية في حالات الطوارئ هو استراتيجية فعالة للحد من العبء العالمي للأمراض. ****تختصر الرعاية في حالة الطوارئ التخصصات التقليدية التي تركز على الأمراض، لتقوم بالتعامل مع مجموعة واسعة من الأمراض والإصابات الحادة التي تسهم بشكل كبير في حالات الوفاة والعجز، وخاصة في البلدان منخفضة ومتوسطة الدخل. ****في حين أن حركة التغطية الصحية الشاملة (UHC) تكتسب الدعم باستمرار، فضلاً عن أن حقوق الإنسان والنظم الصحية هي جزء لا يتجزأ من التغطية الصحية الشاملة، إلا أنه لم تتم إلا عدد قليل من المناقشات الملموسة حول حق الإنسان في الحصول على رعاية الطوارئ حتى الآن. ****علاوة على ذلك، لم يتم اقتراح أي نهج قائم على الحقوق لتطوير أنظمة رعاية الطوارئ. ****نستكشف في هذه المقالة المكونات الرئيسية للحق في الصحة (وهو يعني توفر المنشآت والمتطلبات والخدمات الطبية، وإمكانية الحصول عليها جميعاً ومدى مقبوليتها) نظراً لعلاقتها بأنظمة الرعاية في حالات الطوارئ. ****كما نقترح استخدام إطار عمل قائم على الحقوق للوفاء بالالتزامات الأساسية للحق في الصحة، والتنفيذ التصاعدي للرعاية في حالة الطوارئ في كل البلدان.

## 摘要

提供紧急医疗救援是一个减轻全球疾病负担的有效战略。紧急医疗救援超越传统的、注重疾病的准则，致力于改善导致死亡率和致残率大幅上升的一系列急性疾病和伤害，尤其是在低收入和中等收入国家。虽然全民健康覆盖 (UHC) 运动正在日益获得支持，人权和卫生系统也是全民健康覆盖 (UHC) 中不可或缺的一部分，但迄今为止，在紧急医疗救援领域，很少具体谈及人权问题。此外，尚未提出任何基于人权的方法来发展紧急医疗救援体系。在本文中，我们探讨了与紧急医疗救援体系相关的健康权的关键组成部分（即医疗机构、商品和服务的可用性、可获得性、可接受性和质量）。我们提议，在所有国家使用基于人权的机制来履行健康权的核心义务，并逐步实现紧急医疗救援。

## Резюме

Оказание неотложной помощи — эффективная стратегия в деле снижения глобального бремени заболеваний. В неотложной помощи используются традиционные дисциплины, сосредоточенные на лечении отдельных заболеваний, с целью излечения широкого спектра острых заболеваний и травм, которые ведут к смертности и инвалидности, в особенности в странах с низким и средним уровнем дохода. Несмотря на то что движение за всеобщий охват услугами здравоохранения получает поддержку, а права человека и системы здравоохранения являются неотъемлемой частью всеобщего охвата услугами здравоохранения, до настоящего времени вопросы прав человека и их связи с неотложной помощью почти не обсуждались. Кроме того, отсутствуют предложения по разработке систем неотложной помощи с учетом прав человека. В этой статье авторы рассматривают основные компоненты права на здоровье (то есть физическую доступность, ценовую доступность, приемлемость и качество медико-санитарных учреждений, товаров и услуг здравоохранения) и их связь с системами оказания неотложной помощи. Авторы предлагают заложить представление о правах в основу рамочных схем с целью соблюдения основных обязательств в части права на здоровье и прогрессивной реализации неотложной помощи во всех странах.

## Introduction

Increasingly, the global health community is recognizing the important role that emergency care plays in delivery of health services. Estimations suggest that emergency care could address 54% to 90% of deaths and 900 million to 2.5 billion disability-adjusted life years in low- and middle-income countries.^1^^,^^2^ The effect of emergency care on the burden of disease is due to its ability to deal with a wide variety of acute injuries and illnesses across the lifespan of all populations. While primary prevention efforts are important to reduce the burden of acute diseases, emergencies continue to occur in both the most developed and least developed countries.

Emergency care is a health service that cross cuts traditional disease-focused disciplines and provides prompt interventions for many disease-specific emergencies, including pregnancy-related complications, communicable and noncommunicable diseases and injuries. Health systems in many countries are often fragmented and comprised of programmes with a narrow focus on disease-specific care. However, well organized emergency care appropriately distributed across a country allows for timely coordination of services and resources, and optimum efficiency and efficacy in treating a range of acute conditions, from out-of-hospital care at the scene of an injury or illness to treatment and stabilization in the emergency unit, and early operative and intensive care.^3^ Indeed, emergency care systems address at least 12 of the targets of the sustainable development goals (SDGs; targets 3.1–3.9, 3d, 11.5 and 16.1) and are particularly relevant to universal health coverage (UHC).^4^

Such claims about the effect of emergency care systems are important, but equally imperative is the human rights argument for access to emergency care. A human rights approach to access to emergency care can provide both legal and moral support to advocacy efforts. Legal support relies on a complex collection of international treaties, national constitutions, domestic laws and court rulings pertaining to the so-called right to health. Moral support is philosophically more difficult to define, but no less important for policy-makers and global stakeholders because it bases support for human rights on our shared humanity regardless of existing laws and treaties.^5^

Previous use of a human rights approach to health services has successfully changed global health policy, most notably in the campaign against human immunodeficiency virus (HIV) and acquired immunodeficiency syndrome (AIDS).^6^ By recognizing that stigmatization of people diagnosed with HIV created a marginalized population with reduced access to care, poor health outcomes and unchecked spread of disease, policy-makers were compelled to ensure basic human rights protections.^7^ These protections improved access to health services and medications, guaranteed the availability of preventative measures in vulnerable populations and reduced discriminatory practices.^6^ Such protections have reduced morbidity, mortality and disease transmission; AIDS-related deaths have decreased by more than 51% (0.94 million/1.9 million) since 2004 and new HIV infections by 47% (1.8 million/3.4 million) since 1996.^8^

The focus on vulnerable populations with little access to care and subsequent poor health outcomes has many similarities to the delivery of emergency care. Emergency conditions, such as traumatic injuries, disproportionately affect people in low- and middle-income countries. About 90% of the burden of death and disability from injuries occurs in low- and middle-income countries.^9^ In many parts of the world, vulnerable or marginalized people who are otherwise unable to access health care will seek care for acute conditions and for exacerbations of chronic diseases through their only available means of care, emergency services.^10^

In addition, the urgency of perceived emergency conditions leaves people highly vulnerable to financial pressures. Where access to emergency care is not guaranteed, hospitals may demand exorbitant payment before offering life-saving emergency care, leaving patients and their family members with an impossible decision to make under pressure: pay for life-saving medical care at the expense of housing or food security, or forego care and risk death or permanent disability. In fact, families in many parts of the world are routinely forced to sell assets or borrow money against collateral before care will be provided, and this situation is more likely for households headed by women.^11^^,^^12^ The death of Alex Madaga in Kenya highlights this problem. Mr Madaga sustained serious head injuries from a road traffic crash and died hours later after several health-care facilities had turned away his ambulance. At least two of the facilities denied him admission because his wife could not afford the sizeable deposit.^13^ The injustice of his death shows that access to emergency care can be denied even where emergency services are available.

If emergency care is acknowledged as a human right and the associated obligations this right places on countries are understood, it becomes clear that a nation cannot fulfil its duty to its people without strategically developing emergency care. A rights-based framework for emergency care must therefore: (i) define the legal obligation to respect, promote and protect a universal right to emergency care; (ii) set rights-centred development priorities for emergency care systems in resource-constrained countries; and (iii) provide an instrument to monitor and evaluate emergency care systems considering human rights.^14^

In this article, we explore the foundational arguments for a rights-based approach to emergency care. We review the evolution and key components of the right to health, introduce a rights-based framework for the core obligations that all countries must fulfil to guarantee the right to emergency care, and consider some priorities for the progressive realization of comprehensive emergency care systems.

## Right to health

The right to the highest attainable standard of health has evolved since its first mention in the constitution of the World Health Organization (WHO) in 1946.^15^^–^^17^ Two years later, the United Nations’ Universal Declaration of Human Rights became the first legally-binding treaty to introduce the right to health; it states, “Everyone has the right to a standard of living adequate for the health and well-being of himself and of his family.”^18^

The ratification of the Universal Declaration of Human Rights placed health within the context of human rights for the first time, but offered little direction on what constitutes the right to health. Subsequently, the International Covenant on Economic, Social, and Cultural Rights and the Convention on the Rights of the Child further codified the right to the highest attainable standard of health.^19^^,^^20^ The covenant expresses this right in terms of freedoms (e.g. freedom from medical experimentation without consent) and entitlements (e.g. access to essential medications).^19^^,^^21^ These agreements require nations to respect, promote and protect these rights, and all countries have ratified at least one binding treaty that enforces the right to the highest attainable standard of health.^15^ However, in 2008, fewer than one third of the ratifying countries worldwide had recognized the right to health in their constitutions or national statutes, which is a critical step to full implementation of the ratified treaties.^15^ A study using the Universal Periodic Review (2008–2012) of the Human Rights Council to track implementation of the SDGs noted that 9% (496/5390) of all human rights recommendations from the review concerned health systems and services, but follow-up implementation was low: 21% (32/156) fully implemented and 41% (64/156) partially implemented.^22^

## Right to emergency care

The International Covenant on Economic, Social, and Cultural Rights defined the right to the highest attainable standard of health, but left countries with little guidance on how to promote and protect this right. In response, the Office of the United Nations High Commissioner for Human Rights released General Comment No. 14: The Right to the Highest Attainable Standard of Health in 2000.^23^ This document operationalized the right to health and clarified the scope of countries’ obligations by introducing six core obligations (outlined in the next section) and four interrelated essential elements, availability, accessibility, acceptability and quality (Box 1).^23^

Box 1 **Essential elements of the right to health applied to emergency care**

**Availability**

Definition: Health resources must be available in sufficient quantities within the country to manage the population’s needs, including trained personnel, health-care facilities and essential medicines.

Application to emergency care: Availability of emergency care services requires a sufficient number of emergency units, prehospital and facility-based providers with specific training in emergency care, and essential equipment and medicines, among other things.

**Accessibility**

Definition: Health facilities, goods and services must be distributed in such a way as to be accessible to everyone without discrimination. Special consideration should be given to vulnerable populations, underserved geographic regions and affordability.

Application to emergency care: Accessibility to emergency care depends on coordinated systems that allow patients experiencing acute illness or injury to arrive at a facility that has the necessary capabilities to stabilize the patient or offer definitive care. To make emergency care accessible requires integration of prehospital systems and a coordinated network to transfer patients from basic district hospitals to referral hospitals when needed. Key considerations include coverage in rural and underserved areas, and protection of vulnerable populations (e.g. minorities, indigenous populations, children, pregnant women, refugees and immigrants) from discrimination.

**Acceptability**

Definition: Health facilities and services should be respectful of medical ethics and culturally appropriate to the local context.

Application to emergency care: Emergency care services should be provided in a culturally acceptable manner and be consistent with medical ethics (e.g. treatment of the patient regardless of ability to pay). This obligation requires an open and transparent process in providing and improving emergency care systems that takes account of local customs and needs by encouraging community participation.

**Quality**

Definition: Health facilities, goods and services must be scientifically and medically appropriate and of good quality.

Application to emergency care: Emergency care must be delivered with a focus on quality, which necessitates establishing standards and resource-appropriate best practices, as well as measuring outcomes to ensure quality is met.

Sources: Essential elements;^23^ application to emergency care.^24^^,^^25^

Although not legally binding, General Comment 14 is widely accepted as an authoritative guide to interpreting the right to health.^15^^,^^26^ Numerous court cases concerned with the right to health have been successfully tried in national courts using General Comment 14 as customary practice, which may be enforced as if law.^27^ While other documents and resolutions, such as the SDGs, provide practical targets on certain rights-based topics, they are neither as comprehensive nor enforceable as General Comment 14.

Applied to emergency care, the elements of availability, accessibility, acceptability and quality outline the relevant functions of a health system that are essential to an emergency care system that respects, promotes and protects the right to health. These elements do not represent an exhaustive list of functions that ensure a complete emergency system, but they are useful for setting implementation and funding priorities.

General Comment 14 applies to countries at all levels of economic development. Central to the four essential elements are the overarching concepts of resource availability and progressive realization. These principles mean that developed countries with enough resources are obligated to ensure that the right to health is fully realized, whereas countries with constrained resources are not expected to fulfil this requirement immediately. So as not to permit low-income nations to delay their obligations on the right to health indefinitely, progressive realization means that all nations are required to move “as expeditiously and effectively as possible towards the full realization of article 12” of the International Covenant on Economic, Social, and Cultural Rights.^23^ For example, an advanced nationwide prehospital system with ambulances staffed by trained professionals has not been feasible in Uganda due to resource constraints. Nonetheless, an innovative project that trained police, taxi drivers and community leaders in basic prehospital trauma care could be an effective way of creating a rudimentary prehospital system.^28^ Researchers estimated that if this project was scaled up, it would cost only US$ 0.12 per capita, or US$ 25–75 per life year saved.^28^ This project used available resources to help fulfil Uganda’s core obligations while planning for progressively realizing a more complete prehospital system.

## Core obligations

The core obligations outlined in General Comment 14 are fundamental to the right to health and must therefore be guaranteed immediately, regardless of a country’s economic development; they are important exceptions to the principles of resource availability and progressive realization. Four of the six core obligations relate directly to the delivery of emergency care: (i) access to health facilities, goods and services on a non-discriminatory basis; (ii) provision of essential drugs; (iii) equitable distribution of all health facilities, goods and services; and (iv) adoption and implementation of a national public health strategy and plan of action that addresses the health concerns of the whole population. The remaining two core obligations: (v) access to essential food; and (vi) access to shelter and sanitation, do not directly relate to emergency care. 

Table 1 gives a rights-based framework linked to the core obligations, organized according to WHO’s health system functions,^30^ which countries would have to fulfil when developing emergency care systems.

**Table 1:** Core obligations in General Comment No. 14 relating to emergency care systems

Core obligation	Description		Health system function				
			Leadership and governance^a^	Financing	Human resources and training	Essential medical products technologies and infrastructure	Information and research
**Access**	Ensure the right to access health facilities, goods, and services on a non-discriminatory basis, especially for vulnerable and marginalized populations		Pass laws on access to emergency care without regard to ability to pay, including for migrants and refugees^25^^,^^29^	Fund facilities that provide emergency care for people who cannot otherwise pay^29^	Train providers to recognize emergency conditions and provide initial assessment and resuscitation^25^	Establish an emergency call system with a nationwide number or activation system^29^	Monitor and evaluate access indicators to ensure non-discriminatory practices^29^
			Mandate initial screening and stabilization of a patient before any payment is required^25^		Create protocols for triage and emergency stabilization before registration^25^^,^^29^		
			Create legal protections for good Samaritans^25^^,^^29^		Train staff on delivery of emergency care according to need alone^25^		
**Essential medicines**	Provide access to essential medicines		Pass laws guaranteeing access to essential medicines^29^	Regulate the pharmaceutical market for essential medications^29^	Train pharmacists and clinicians on transfusion and safe administration of medicines^25^	Ensure availability of the WHO’s Model Lists of Essential; Medicines^25^ and the essential emergency medication list of AFEM^25^	Monitor and evaluate availability of emergency medicines^29^
**Equitable distribution**	Ensure equitable distribution of health facilities, goods and services		Establish emergency referral networks and transport protocols^25^	Finance facilities in regions with limited access	Train lay and professional responders in emergency care^25^	Ensure the availability of communications technology for out-of-hospital emergency care and between facilities^25^	Measure and evaluate equitable distribution of emergency care services
			Certify capacity of emergency facilities^25^				
			Distribute care centres with specialized services across the country^25^	Finance transport systems	Distribute providers trained in emergency care across the country^25^^,^^29^		
**National public health strategy**	Adopt and implement a national public health strategy and plan of action		Create a national plan to develop emergency care systems^29^	Create a national plan for financing universal access to emergency care^29^	Provide bystander and community-based training on first aid, system activation, and care-seeking behaviour^25^	Use a registry platform for all targeted emergency conditions (including trauma)^4^	Adopt syndromic surveillance guidelines in emergency units^25^
			Designate an emergency care liaison to the national public health office^25^				
			Coordinate national disaster preparedness with emergency facilities and providers of out-of-hospital emergency care^25^		Train community and health-care providers on disaster preparedness and response^25^		Institute requirements for quality improvement procedures based on process and outcome performance data^25^
			Develop community-based response regulations				

AFEM: African Federation for Emergency Medicine.

^a^ Governance includes legislation, regulation and protocols that require the delivery of emergency care.^25^

Notes: The table presents four out of the six core obligations in General Comment 14^23^ that relate directly to the delivery of emergency care. The two other core obligations are (i) access to essential food; and (ii) access to adequate shelter and sanitation. Good Samaritan is a bystander to an accident or illness who provides assistance in some form.

### Access

Countries have an obligation to ensure the right to access health facilities, goods and services on a non-discriminatory basis. The obligation to protect from discrimination is particularly important for vulnerable or marginalized groups. All governments should therefore create legislation that guarantees access to emergency care services for all people regardless of race, ethnicity, religion, citizenship status or ability to pay. For example, the constitutions of South Africa (Article 27)^31^ and Kenya (Article 43)^32^ guarantee that no one may be refused emergency medical treatment, while legislation in the United States of America (Emergency Medical Treatment and Active Labour Act)^33^ mandates that anybody who presents to an emergency department for care must be screened and stabilized before requesting payment.

### Essential medicines

Essential medicines are those that “satisfy the priority health care needs of the population.”^34^ These medicines should be available in sufficient quantities and with assured quality at all times.^35^ A government’s duty to provide access to essential medicines is already enshrined in several national constitutions.^36^ A study in 2006 reported 59 court rulings from low- and middle-income countries in which access to essential medicines was successfully claimed under the right to health.^27^ Timely access to essential medicines during an emergency is a key function of emergency care systems. This requirement recently prompted the African Federation for Emergency Medicine to develop a list of essential medicines specifically for the delivery of quality emergency care.^37^

### Equitable distribution

All countries are obligated to ensure equitable distribution of health facilities, goods and services. For emergency care systems, this obligation requires a specific plan that distributes specialized services equitably between regions of a country, coordinates referral networks and places trained providers in the locations where the population needs them. Population-level spatial analysis for prehospital systems has been shown to be a feasible method of understanding the geographic prehospital needs of the population in Ghana.^38^

The same geospatial approach can be used for both planning the positioning of facilities for treating emergency conditions and assessing the current distribution of facilities to identify any mismatch with population needs.^39^^,^^40^ However, this approach is of limited use in settings where the emergency care capabilities of each facility are unknown. Researchers have noted that proximity to a hospital does not guarantee access to emergency care, since many facilities in low- and middle-income countries lack the trained staff and resources necessary to deliver good-quality emergency care.^41^ In addition, marginalized populations, such as migrants or refugees, may not be located where populations are densest (e.g. cities). Thus, a system that primarily considers population density may neglect to provide adequate, non-discriminatory access to vulnerable populations.

### National public health plan

A national public health strategy cannot be complete without inclusion of an emergency care system. These systems are important not only for everyday public health needs, but also for maintaining resilient health systems that are capable of responding to disasters, disease outbreaks and other crises.^4^ The process of developing and refining the national health plan must be transparent and participatory to ensure both its appropriateness and quality, as highlighted in the Declaration of Alma Ata and Ouagadougou Declaration as they pertain to primary health-care systems.^42^^,^^43^ While the emergency care system is concerned with the acute phases of an accident or illness, it is also an important point of access for many people seeking care who may then be referred to rehabilitation or primary health care follow-up.^44^

## Progressive realization

The core obligations are the foundation of a rights-based emergency care system, but progressive realization drives most of the ongoing development and refinement of the system. Once countries have fulfilled the core obligations, they must work quickly and effectively to fully achieve the right to emergency care. As a result of of the complexity of emergency care systems and differences in country contexts and resource availability, a single pathway for development of such systems that is appropriate for all countries does not exist. However, the four essential elements set out in General Comment 14 can help prioritize the development of each component of the emergency care system. Indeed, 15 years after the release of General Comment 14, the right to health is still a priority in the 2030 agenda for sustainable development. While the agenda is not a binding human rights document, the targets of its SDGs are based on human rights and feature prominently the principles of equality and non-discrimination.^22^

Examples of progressive realization can be found in components of the emergency care system. In out-of-hospital emergency care, timely care at the scene of an injury or illness and prompt transport to a health-care facility save lives. Out-of-hospital emergency care is an important access point to the emergency care system. However, the prehospital system, including trained providers (e.g. paramedics) and ambulances, which is common in high-income countries, is too costly for most low- and middle-income countries. Instead, Iraq, Cambodia and South Africa successfully introduced lay first responders, drawn from the community, at a lower cost.^45^^,^^46^ As resources allow, the emergency care system should be expanded to include professional prehospital responders. Implementing certification of emergency medical technicians in Mexico nearly halved the risk of death in people treated by this emergency care service.^47^

Delivery of good-quality emergency care requires a health workforce with training in emergency care. While many high-income countries have a full team of physicians and nurses specialized in emergency medicine, low- and middle-income countries may rely on clinical officers, independent nurses and general practice physicians to provide frontline emergency care. Therefore, training of these health-care staff is important. For example, training of staff in a dedicated paediatric emergency area in Malawi to perform emergency triage assessment and treatment halved inpatient mortality.^48^ In the Democratic Republic of the Congo, training non-specialists to perform correct, basic orthopaedic care of open fractures reduced amputation rates, from 100% to 21%.^49^ Through a public–private partnership that mobilized sufficient resources in the United Republic of Tanzania, Muhimbili National Hospital launched the country’s first emergency medicine residency programme to train specialist doctors.^50^ These examples demonstrate that gradual improvements are feasible and in keeping with the concepts of resource availability and progressive realization.

## Assessing progress

The use of a rights-based approach is not only important during the development of emergency care systems, but also for evaluating and improving to the system. Assessment is essential to ensure that countries are accountable and meet their human rights obligations.^51^ Assessment should include indicators of health and human rights that help governments and non-state actors measure progress and identify gaps. Monitoring at the global level (e.g. through the Universal Periodic Review) can track progress and allow planning for the progressive realization of emergency care in individual nations.^22^

Health and human rights indicators are most often either health indicators that draw conclusions about human rights promotion, or human rights indicators that indirectly measure health outcomes.^52^ Recently, a hybrid of health and human rights indicators has emerged, which looks at the existence of health-related laws and regulations, their quality and their implementation.^52^ Regardless of which type of indicator is used, specific indicators for the promotion and protection of the right to emergency care should be drawn from the four essential elements. Special attention must be paid to the quality of care delivered, protection of vulnerable populations, involvement of the community, transparency, methods for obtaining indicator data, and the intended use of the results to avoid unintentional violations of the rights of certain groups during the assessment process.^52^^,^^53^

## Conclusion

Emergency care is an often overlooked, but essential component of the right to the highest attainable standard of health and UHC. Particularly for vulnerable and disadvantaged populations, emergency care is often the last chance for the health system to save a life. In view of the obligations placed on governments to respect, promote and protect the right to the highest attainable standard of health, countries must prioritize the funding and implementation of emergency care systems. International organizations such as the United Nations, WHO and the World Bank should be tasked with providing the technical guidance for countries to implement a rights-based framework for emergency care and following through with monitoring and evaluation. Implementation of a rights-based framework for emergency care requires countries to enact legislation that ensures access to non-discriminatory emergency care and establish a regulatory body with appropriate oversight and authority to enforce these laws.

All countries, regardless of resources and economic development, must begin by ensuring that the core obligations are fulfilled. Once these obligations are met, countries should use the essential elements in General Comment 14 to progressively build a comprehensive emergency care system and should continuously evaluate progress. The call for countries to develop and improve emergency care systems is justified not only by the positive effect emergency care will have on the well-being of the population, but also by the obligation to respect, promote and protect the right to the highest attainable standard of health. We argue that this obligation cannot be fulfilled without a rights-based approach to provision of good-quality emergency care.
